# Belowground Response to Drought in a Tropical Forest Soil. II. Change in Microbial Function Impacts Carbon Composition

**DOI:** 10.3389/fmicb.2016.00323

**Published:** 2016-03-15

**Authors:** Nicholas J. Bouskill, Tana E. Wood, Richard Baran, Zhao Hao, Zaw Ye, Ben P. Bowen, Hsiao Chien Lim, Peter S. Nico, Hoi-Ying Holman, Benjamin Gilbert, Whendee L. Silver, Trent R. Northen, Eoin L. Brodie

**Affiliations:** ^1^Climate and Ecosystem Sciences, Earth and Environmental Sciences, Lawrence Berkeley National LaboratoryBerkeley, CA, USA; ^2^International Institute of Tropical Forestry, United States Department of Agriculture Forest ServiceRio Piedras, PR, USA; ^3^Fundación Puertorriqueña de ConservaciónSan Juan, PR, USA; ^4^Environmental Genomics and Systems Biology, Life Sciences Division, Lawrence Berkeley National LaboratoryBerkeley, CA, USA; ^5^Department of Environmental Science, Policy, and Management, University of California, BerkeleyBerkeley, CA, USA

**Keywords:** tropical forest, drought, microbial functions, carbon dioxide, soil carbon

## Abstract

Climate model projections for tropical regions show clear perturbation of precipitation patterns leading to increased frequency and severity of drought in some regions. Previous work has shown declining soil moisture to be a strong driver of changes in microbial trait distribution, however, the feedback of any shift in functional potential on ecosystem properties related to carbon cycling are poorly understood. Here we show that drought-induced changes in microbial functional diversity and activity shape, and are in turn shaped by, the composition of dissolved and soil-associated carbon. We also demonstrate that a shift in microbial functional traits that favor the production of hygroscopic compounds alter the eﬄux of carbon dioxide following soil rewetting. Under drought the composition of the dissolved organic carbon pool changed in a manner consistent with a microbial metabolic response. We hypothesize that this microbial ecophysiological response to changing soil moisture elevates the intracellular carbon demand stimulating extracellular enzyme production, that prompts the observed decline in more complex carbon compounds (e.g., cellulose and lignin). Furthermore, a metabolic response to drought appeared to condition (biologically and physically) the soil, notably through the production of polysaccharides, particularly in experimental plots that had been pre-exposed to a short-term drought. This hysteretic response, in addition to an observed drought-related decline in phosphorus concentration, may have been responsible for a comparatively modest CO_2_ eﬄux following wet-up in drought plots relative to control plots.

## Introduction

Tropical forests have a disproportionate capacity to affect Earth’s climate relative to their areal extent. Despite covering only 12% of land surface, tropical forests account for ∼35% global net primary productivity (Phillips et al., 1998; Grace et al., 2001), and are among the most significant of terrestrial carbon (C) stores (Jobbágy and Jackson, 2000; Artz et al., 2008; Pan et al., 2011). The relatively consistent warm and humid conditions of tropical forests promote high rates of net primary productivity (Grace et al., 2001), which, despite high microbial decomposition (Raich et al., 2006), render the soils either net C sinks or stable in size (Phillips et al., 2008). However, predicted changes to the frequency and magnitude of precipitation (Burke and Brown, 2008) suggest an increased risk of drought in tropical regions that could alter the C balance (Phillips et al., 2009, 2010; Gatti et al., 2014; Doughty et al., 2015), and potentially feed back to atmospheric carbon dioxide (CO_2_) concentrations and climate (Townsend et al., 2011).

Soil moisture is a key driver of belowground biogeochemical processes (Rodriguez-Iturbe and Porporato, 2005). A reduction in soil moisture under drought can alter preferential flow paths of water through porous networks in mineral soils, disrupt water film continuity and constrain the diffusion of extracellular enzymes and substrates (Or et al., 2007; Manzoni and Katul, 2014). Drought can also concentrate solutes within remaining water films, reducing the water potential of the physical matrix and imparting osmotic stress to soil biota (Schimel et al., 2007; Moyano et al., 2013). Furthermore, within the clay rich mineral soils common to tropical regions, soil moisture plays a deterministic role in soil oxygen and redox dynamics (Yu et al., 2006; Liptzin et al., 2010; Silver et al., 2013). This is significant because an increase in soil oxygen as moisture declines can trigger the precipitation of soluble ferrous iron to ferric iron complexes, and the simultaneous co-precipitation of organic matter and phosphorus (Kleber et al., 2015), forming a strong geochemical C and nutrient sink (Silver et al., 1999; Baldock and Skjemstad, 2000; Lalonde et al., 2013). These changes in the physical environment feed back not only on the phylogenetic and functional composition of microbial communities (Bouskill et al., 2013), but also their activity (Manzoni et al., 2012; Bouskill et al., submitted), and the rate of soil biogeochemical processes (Manzoni and Katul, 2014), and consequent trace gas fluxes (Wood and Silver, 2012).

The phylogenetic and functional response of microbial communities under drought are an emergent property of the ecosystem determined by life history strategies and traits (Lennon et al., 2012; Evans and Wallenstein, 2014) filtered by the environment (Lebrija-Trejos et al., 2010; Crowther et al., 2014; Freedman and Zak, 2015). Previous studies have established that functional traits are non-randomly distributed across phylogenies (Martiny et al., 2012; Goberna and Verdú, 2015), and that this dissemination is, in part, determined by ecosystem properties (Lennon et al., 2012). However, the significance at the biogeochemical scale of trait redistribution following changes in community composition is poorly understood.

Indeed, for microbes performing relatively ‘broad’ (sensu, Schimel and Bennett, 2004) functions, such as the decomposition and turnover of organic matter, a reorganization of functional gene diversity (i.e., the functional potential of a community) does not necessarily lead to changes in an ecosystem function, such as decomposition rate (Allison and Martiny, 2008; Miki et al., 2014). However, an ecosystem perturbation that modifies microbial community structure can also alter intracellular allocation pathways within organisms and populations (Lennon et al., 2012; Schimel and Schaeffer, 2012; Manzoni et al., 2014). Microorganisms reallocate carbon reserves to osmolyte production under declining water potential to maintain cellular turgor and function (Welsh, 2000; Schimel et al., 2007). Similarly, constraints on substrate diffusion in drying soils can promote the allocation of carbon to extracellular enzyme production, stimulating the depolymerization of plant-polymers to readily acquirable monomers (Zeglin et al., 2013). In this way, the competitive dynamics of the microbial community that lead to individual groups of microorganisms modifying their intracellular allocation pathways, tied to life history traits, can shape the composition of soil carbon (Chenu and Cosentino, 2011; Liang and Balser, 2011; Miltner et al., 2011; Schimel and Schaeffer, 2012). Consequentially, the environmental selection of microorganisms as a function of their allocation and growth strategies, i.e., their ‘response traits,’ can feed back to alter the distribution and expression of traits that directly impact their environment, such as extracellular enzymes that alter stability and flux of soil carbon soil, i.e., their ‘effect traits’ (Lavorel and Garnier, 2002; Martiny et al., 2015).

Seasonal precipitation dynamics in arid and semi-arid ecosystems can result in pulsed activity events where large eﬄuxes of CO_2_ from soil are observed upon the first rainfall after drought (Xu et al., 2004). A number of factors contribute to this pulse that is known as the Birch effect. These include physical disruption of soil that improves microbial access to formerly occluded organic carbon; the release and metabolism of microbial osmolytes; or the enhanced mineralization of accumulated depolymerized substrates. In tropical soils, a phenomenon similar to the Birch effect has also been observed (Liptzin and Silver, 2015), and is sustained by redox reactions in the clay-rich mineral soils. Oxidizing conditions in these soils promotes the sorption and/or co-precipitate of organic matter and nutrients to iron minerals and clays (Kleber et al., 2015). These compounds can then be released by microbial reductive metal dissolution under the oxygen limited conditions that occur following rainfall events (Liptzin and Silver, 2015). This release of previously inaccessible organic C and the availability of alternate electron acceptors combined with a population of microorganisms adapted to these redox oscillations (Pett-Ridge et al., 2006; Dubinsky et al., 2010), could sustain the magnitude of these pulsed-activity events. On the other hand, microorganisms under water potential or oxidative stress are known to increase production of extracellular polysaccharides (EPS, O’Toole and Stewart, 2005), encasing themselves within hygroscopic biofilms that retain moisture within the matrix as soils dry (Chenu, 1993), widening the water potential niche within which an organism grows optimally (Lennon et al., 2012). Biofilms therefore buffer microbial physiology from rapid environmental changes in water potential, redox, and substrate availability (Or et al., 2007; Holden, 2011). Moreover, the enhanced stability afforded by EPS production can alter the soil structure (Chenu and Cosentino, 2011) and reduce the osmotic shock preceding cell lysis that typically occurs during rewetting of dry soils in arid and semi-arid systems (Fierer and Schimel, 2003). In this way the magnitude of CO_2_ pulses following re-wetting may be impacted positively by traits related to osmolyte or extracellular enzyme production, and negatively by traits related to biofilm production.

### Study Site Description, Recent Research, and Current Objectives

Here we leverage a precipitation-manipulation experiment designed to systematically evaluate the impact of drought on microbial community functional potential and activity, to examine the consequences for the carbon cycling in humid tropical forest soils.

#### Site Description and Throughfall Exclusion Experiment

The experiments were conducted by reducing the through-canopy precipitation (throughfall) in a humid tropical forest in the Bisley watershed (181.18N, 65.50W) of the Luquillo Experimental Forest (LEF), Puerto Rico. The site is ∼350 m above sea level and receives ∼3500 mm of precipitation per year, and ∼200 mm per month. The detailed classification of these soils has been published previously (Dubinsky et al., 2010), but briefly, the soils are classified as ultisols in the Humatus-Cristal-Zarzal series, and are derived from volcanic sediments with Tertiary-age quartz-diorite intrusions of the Rio Blanco stock. The soils are deep, clay rich and acidic, with high aluminum and iron content (Scatena, 1989).

The throughfall exclusion experiment was established in a Tabonuco forest stand located on an upper ridge. Throughfall was excluded with clear, corrugated plastic panels (1.6 m^2^) mounted 1 m above the forest floor at a 17° angle. Ten plots were established randomly between tree stands in June 2008, and five of these excluded for a period of 3 months before the shelters were removed and ambient throughfall resumed (in the present study these plots are termed pre-excluded). The following year (June, 2009), the shelters were replaced over the original treatment plots and five new exclusion plots were established (termed *de novo* in the present study), the remaining five plots were never sheltered and served as control plots. The plots were not trenched to minimize soil disturbance and allow root interaction and leaf litter was manually replaced from the top of the shelters back to the soil beneath every 4–7 days. As a consequence, organic carbon concentrations in the throughfall excluded soil plots did not fall over the course of the experiment and were not significantly different to the control soils (Bouskill et al., 2013). Soil samples (5–10 g) were taken in triplicate to a depth of 8–10 cm using soil corers 10 months following the placement of the throughfall shelters. Samples were shipped to Berkeley overnight on ice and the triplicate samples composited immediately. Fresh material was allocated for anion/cation analysis and metabolite profiling, with the rest of the soil frozen at -80°C prior to spectroscopy.

#### Previous Research

In the present study, 10 months of drought treatment through throughfall exclusion led to a small decline in soil water potential (from of -0.19 MPa to -0.34 MPa). Despite this, and with no observed difference in microbial biomass between control and treatment plots, the bacterial phylogenetic diversity in *de novo* plots decreased 40% compared with the control plots. Pre-excluded plots showed no change in diversity metrics. Conversely, the relative abundance of different bacterial taxa in the pre-excluded and *de novo* plots changed significantly toward phyla associated with deep-branching response traits for declining water potential (e.g., Actinobacteria, Bouskill et al., 2013). Such a profound response in community composition might be indicative of limited intra-species adaptive capacity to a rarely experienced perturbation and perhaps symptomatic of relatively aseasonal wet tropical ecosystems (Deutsch et al., 2008). A follow-up study noted a coupling between the functional potential of the microbial community and the expression of that potential (Bouskill et al., submitted). Communities under drought (both pre-excluded and *de novo*) displayed a common response to drought stress: elevated abundance of genes related to oxidative and osmotic stress, and the increased synthesis of common intracellular osmolytes (trehalose, ecotone; Potts, 1994; Welsh, 2000).

Soils undergoing drought also had a greater abundance of genes related to the degradation of complex plant polymers, along with higher activities of enzymes related to the breakdown of cellulose and hemicellulose (e.g., β-glucosidase, cellobiohydrolase, and xylanase). We therefore hypothesized that small changes in Ψ can select for a microbial community composition capable of responding to osmotic stress via osmolyte production. Such a response requires the intracellular reallocation of C that likely drives an overall elevated C demand (Manzoni et al., 2014). This demand could, in turn, prompt the measured increase in enzyme activity and a potential decomposition of plant-derived polymers. However, the implication for the composition of soil carbon, and its fate following the resumption of precipitation is poorly understood.

Soils experiencing repeat perturbation (i.e., the pre-excluded soils) showed a more modest response, geochemically, hydrologically, and biologically, to prolonged drought. The specific mechanism behind this resilience has yet to be characterized, however, this suggests that the adaptive capacity of bacteria in this humid tropical forest could widen under repeat perturbation.

#### Current Objectives

In this manuscript, we leverage the LEF rainfall manipulation experiment described above to examine the impact that previously characterized changes to the functional diversity of microbial communities (Bouskill et al., submitted) under drought might have on the carbon cycle of a humid subtropical forest soil. We address three specific questions: (1) do changes in the functional potential of perturbed microbial communities feed back onto the composition of soil carbon? (2) Do pre-excluded soils exhibit shifts in carbon composition at the molecular scale that signify a mechanism for resilience to prolonged drought? (3) Do changes in soil carbon composition relate to the magnitude of carbon lost under simulated conditions of wet-up from precipitation relative to soils not exposed to drought?

## Materials and Methods

### Dissolved Organic Carbon (DOC) Separation

The Dissolved organic carbon (DOC) profile of soil communities was reconstructed to examine changes in community function at the highest level of functional expression. The high soil clay content did not allow for direct extraction of porewater. Therefore, soil water was extracted from 15 g of fresh soil by adding 15 ml ultra-pure water and vortexing (10 min). Metabolites were extracted following previously published protocols (Baran et al., 2010, 2015). Polar compounds were extracted by adding 1 ml methanol (-20°C) to 1 ml of the solution, vortexing for 30 s and incubating at -20°C for 3 min. Following incubation the solution was centrifuged for 1 min at 2,350 *g* and 750 μl removed to a glass vial. Non-polar metabolites were extracted by adding 500 μl hot isopropanol (65°C) to the solution and incubating at 65°C for 3 min. The solution was centrifuged for 14,000 *g* and 750 μl removed to a glass vial. Both polar and non-polar supernatants were concentrated by spinvac and redissolved in 100 μl of methanol containing an internal standard, 1 μg ml^-1^ of 2-amino-3-bromo-5-methylbenzoic acid. The samples were stored at 4°C, filtered through a 0.2 μm PVDF membrane microcentrifugal filter (National Scientific) and analyzed via LC-MS using normal phase liquid chromatography (ZIC-HILIC capillary column, Agilent 1200 series capillary LC system) coupled to a quadrapole time-of-flight mass spectrometer (Agilent 6520 dual-ESI-Q-TOF). Run in positive and negative mode, this method gave signal intensity and spectra data across a wide range (m/z range 52.08–1663.03) from each of the 15 samples. MassHunter software (Agilent, Santa Clara, CA, USA) was used to define and quantify the raw spectra before uploading to MZmine (Chevin et al., 2010; Pluskal et al., 2010; Kellermann et al., 2012) for untargeted identification of spectra. This approach was also used to compare the DOC profile of control vs. drought soils and quantify the extent of shared or unique features in control and treatment soils. Features were identified by spectral comparison to online databases, (MassBank^1^, KEGG^2^). On occasions where more than one potential compound was returned, the compound with the lowest mass difference was retained. The beta-diversity of DOC profiles was visualized by extracting the signal intensity of each feature and analyzing the data in a manner similar to that described previously for functional gene data (Bouskill et al., submitted). Prior to analysis, the metabolite spectra data were manually edited to remove poorly replicated features, log-transformed and converted into a weighted distance matrix using χ^2^ distance (using the R package, labdsv; Roberts, 2007). Several approaches were employed to examine how throughfall exclusion affected the functional diversity of subtropical soils. (1) Mantel tests were initially performed to test for correlations between intersample distances in the metabolite dataset with our previously published intersample phylogenetic distance dataset from the same experiment (Bouskill et al., 2013). (2) Community ordinations were used to project the spatial dissimilarity between control and the exclusion soils. (3) Variance partitioning methods (permutation multivariate anova) were applied to determine the proportion of observed changes in the metabolite datasets that could be related to physico-chemical factors. (4) Canonical correspondence analysis (CCA) linearly correlating environmental variables with biological variables was used to link the metabolite profile structure with an array of environmental variables. Finally, false discovery-corrected ANOVAs (Holm and BH) identified the most significant compounds contributing to the main differences between control and treatment soils and the pre-excluded and *de novo* soils (**Figure 2**).

### Fourier Transform Infrared Spectroscopy

The infrared spectra of soil samples were obtained using attenuated total reflectance (ATR) Fourier Transform Infrared (FTIR) spectroscopy. Frozen soil samples were thawed at room temperature in a chamber filled with inert Argon gas before being gently pressed down on a clean surface of the Germanium crystal in an ATR configuration (Smart Orbit, Thermo Fisher). Infrared light beamed from the interferometer (Nexus 870, Thermo Nicolet) was focused onto the interface between the soil sample and the top surface of the crystal. The sample spectrum was recorded with a spectral resolution of 4 cm^-1^ and a spectral peak position accuracy of 0.01 cm^-1^ over the infrared range (400–4,000 cm^-1^). All spectra were vector-normalized in the biochemical fingerprint region between 1200 and 500 cm^-1^, subject to multivariate principal components analysis (PCA) and linear discriminant analysis (LDA), according to a previously described approach (Deutsch et al., 2008; Hu et al., 2012). Briefly, PCA and LDA were used to generate new variables (factors) that were linear combinations (i.e., weighted sum) of the original variables (wavenumbers). PCA was applied to the spectra to reduce the hundreds of absorbance intensities at different wavenumbers to a few factors that capture >95% of the variance. We selected nine components based on the 95% of variance explained and the spectral features on the loading plot. LDA was applied to maximize ‘inter-class’ variance over the ‘intra-class’ variance of the factors. We visualized the data in the form of score plots and cluster vector plots.

### Excitation-Emission Matrix (EEM) Fluorescence Spectroscopy

Optical analyses of soil carbon solutions provides an approach for comparing the concentration and composition of DOC (Coble et al., 2014). Soils in the Tabonuco forest contain goethite and kaolinite minerals common to ultisols that effectively bind C to reactive mineral surfaces, potentially an important C sink under drought. We extracted any mineral bound C using a pyrophosphate solution. 1 g of soil was added to 40 ml of 100 mM solution of sodium pyrophosphate (pH = 10) and shaken for 4 h, before centrifugation for 15 min (10,000 *g*). For optical analysis, an aliquot of the extraction solution (<10 μL) was added to 3 mL of 0.2 M sodium phosphate buffer at pH 7.0 in a 1 cm path length clean quartz cuvette and mixed thoroughly. Ultraviolet-visible (UV-Vis) absorption spectra were acquired using an Ocean Optics 4000 spectrometer (Dunedin, Florida). Following this, the optical excitation-emission-matrices (EEMs) were acquired on samples diluted in 0.2 M phosphate buffer using a Horiba Fluorolog-3 spectrofluorometer (Kyoto, Japan). Difference EEMs (δEEM) were generated to reveal pairwise differences between samples (Huey et al., 2009; Yan et al., 2013). We implemented a reproducible and automated algorithm to generate δEEMs using a non-linear least-squares (Levenberg-Marquardt) algorithm to find the best value of a scale factor that minimized the sum of the squares of the intensity differences between one unscaled (EEM_1_) and one scaled EEM (αEEM) at each valid λ_ex_/λ_em_ data point. δEEM was estimated using, _δEEM = EEM_1_-αEEM_.

### Laboratory Simulated Throughfall Resumption Experiments

We used laboratory microcosm experiments to examine how the resumption of precipitation following drought might impact CO_2_ flux from treatment and control soils in the presence and absence of supplemental carbon and nutrients. We collected ∼500 g of soil down to the same depth as above (∼8–10 cm) from each of the 15 plots (control, pre-excluded, *de novo*) in June 2010, one full year following shelter placements, and transported them overnight to the laboratory. Upon arrival 75 g of soil from each soil plot was added to mason jars and allowed to equilibrate for 1 week (*T* = -168 h). We measured CO_2_ flux under the resumption of throughfall via the addition of (i) distilled water alone, and (ii) water with litter-leachate solution (concentration = 1629 μgC ml^-1^), made according to previous studies (Cleveland et al., 2004; Wieder et al., 2008). In these soils, litter is a significant source of dissolved organic matter and nutrients to the soils. For the wet-up experiment water was added to the soils at a volume sufficient to maintain the original water content of the different soils (average = 4.3 ml ± 0.8 ml). For the wet-up ± leachate experiments, we added enough litter-leachate solution to simulate 1-weeks’ worth of litter carbon based on the long-term average of litterfall C inputs for the study-site (Lodge et al., 1991). Following the addition of the litter-leachate solution, sufficient deionized water was added to maintain field moist conditions. Thus, approximately 1.1 ml of the leachate/deionized water was added to the control soils and 1.5 ml to the treatment soils. Following wet-up ± litter leachate (*T* = 0 h) the jars were capped and 30 mL air samples were collected with an air-tight syringe and injected into pre-evacuated 20 mL glass vials fitted with Geo-Microbial septa (GMT, Ochelata, OK, USA). Samples were analyzed by gas chromatography (GC) on a Shimadzu GC-14A (Shimadzu Scientific Inc., Columbia, MD, USA), equipped with a Porpak-Q column, using a thermal conductivity detector (TCD) for CO_2_ detection. Samples were subsequently collected at *T* = -168, 1, 4, 24, and 72 h, and then once a week for the following 3 weeks (*T* = 168, 336, and 504 h). CO_2_ fluxes were calculated from the concentration change over time, and were determined using an exponential curve-fitting procedure (iterative model) described by Matthias et al. (1978). Fluxes were considered to be zero when the relationship between time and concentration was not significant at *p* = 0.05.

## Results

### Impact of Drought on Soil Organic *C*-Pools

**FIGURE 1:**
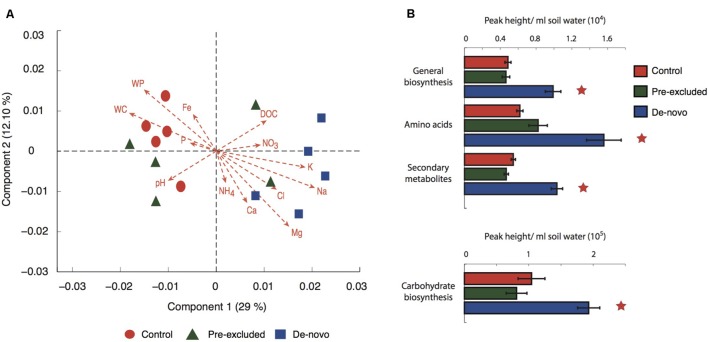
**Changes in metabolomic profiles in treatment vs. control soils. (A)** Weighted canonical correspondence analysis (CCA) of the complete set of metabolites produced by LC-MS^2^ analysis. The ordination is the product of a correlation of functional diversity with physicochemical factors (abbreviations: NH_4_, ammonia; NO_3_, nitrate; K, potassium; Na, sodium; Mg, magnesium; WP, water potential; WC, water content; Fe, iron; P, phosphate; DOC, dissolved organic carbon; Ca, calcium). **(B)** Abundance of selected broad classes of metabolites. Stars above the plots denote significant differences when compared with the control.

Using LC-MS/MS mass spectrometry (environmental meta bolomics), Fourier Transform Infrared Spectroscopy (FTIR) and Excitation Emission Matrix fluorescence spectroscopy (EEM) we assessed the effects of drought on the composition of the DOC pool and mineral associated compounds. Ordination of the complete suite of LC-MS spectral features (∼8,500 features) demonstrated a separation of the control soils from the *de novo* treatment soils across the primary axis (**Figure 1A**). The pre-excluded soils were more dispersed. CCA analysis demonstrated that the DOC composition across all soils was significantly related to soil water solute concentrations (**Figure 1A**). A non-parametric analysis of variance showed that Na, and the interaction between Na and P, explained approximately 33% of the variance in the DOC dataset (Supplementary Table S2B).

The majority of DOC features were common to all soils with the control and pre-excluded soils sharing, on average, 89% of the features within a twofold margin of difference in peak area. The DOC composition of the control and *de novo* drought soils were slightly less similar, sharing on average 84%. Grouping the DOC into discrete classes showed a significantly higher abundance (*p* < 0.05) of all the classes (general biosynthesis, amino acids, secondary metabolites, and carbohydrate biosynthesis) within the *de novo* soils (**Figure 1B**).

**FIGURE 2:**
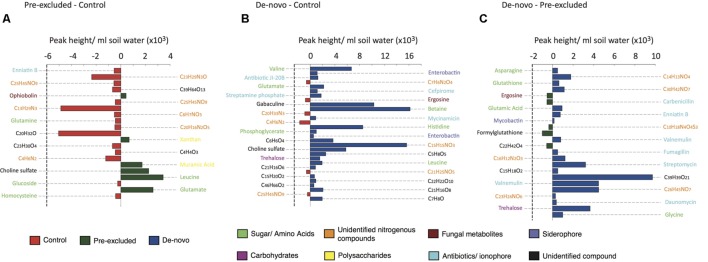
**Comparative abundance and putative identification of the top 20 key metabolites statistically differentiating the control and drought sites.** Individual panels show the pairwise analysis between **(A)** control plots and pre-excluded plots, **(B)** control plots and *de-novo* plots, and **(C)** pre-excluded plots and *de-novo* plots. The bars are coded by site (control, re-excluded, and *de novo*), and the broad classes of the different compounds are color-coded.

Only compounds that could be putatively named or identified at the molecular formula level were included in the final analysis (see Supplementary Table S4 for the full list). Of the DOC features discriminating the control from treatment soils, biochemical signatures of osmotic stress and microbial secondary metabolism were amongst the most significant. The majority of the compounds discriminating control from pre-excluded soils were only identifiable at the molecular formula level (**Figure 2A**). The few compounds elevated in pre-excluded soils were mainly polysaccharides (e.g., xanthan) and amino acids (e.g., leucine).

Comparison of the control and *de novo* soils showed the majority of discriminatory compounds to be higher in the *de novo* soils (**Figure 2B**). These compounds included amino acids (valine, glutamate, leucine) and carbohydrates (trehalose) that might reflect osmotic stress induced by drought (Bouskill et al., submitted). Antibiotics, including fungal and Actinobacterial derived compounds, were either significantly higher in concentration (*P* < 0.05), or solely found, in the *de novo* soils compared with the controls. Higher siderophore abundance in the *de novo* soils possibly reflects a recorded decline in soluble Fe and Mo concentrations (Supplementary Table S1). Similarly, higher abundances of antibiotics and amino acids in the *de novo* soils and the higher abundance of unidentified nitrogenous compounds in the pre-excluded soils accounted for the main differences between the two treatment soils.

Furthermore, we found a significant correlation between the composition (but not concentration) of the DOC pool (as inferred through metabolomics) and enzyme activity (Supplementary Table S2. Activity data for β-glucosidase, Cellobiohydrolase, *N*-acetyl-D-glucosaminidase, and Xylanase presented in Bouskill et al., submitted), suggesting a functional link between the observed microbial metabolic activity and soluble C composition. This was further supported by a positive correlation between both the phylogenetic (Bouskill et al., 2013) and functional potential (Bouskill et al., submitted) of the soil microbial communities and DOC composition (Mantel. Phylogenetic∼DOC: *r* = 0.61, *p* = 0.048. Functional∼DOC: *r* = 0.5, *p* = 0.02. Supplementary Table S3).

### Fourier-Transform Infrared Spectroscopy

**FIGURE 3:**
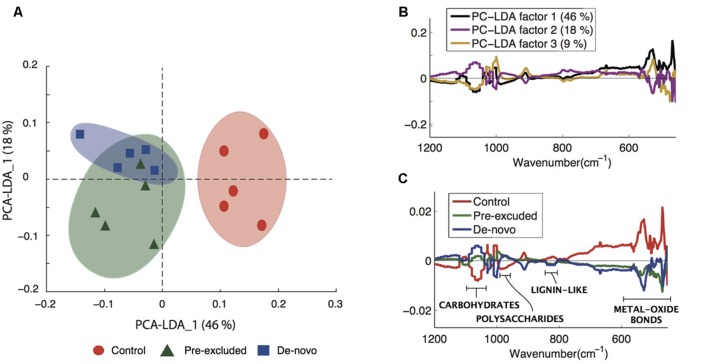
**Changes to carbon composition in treatment vs. control soils. (A)** principal components analysis (PCA)-linear discriminant analysis (LDA) of the complete Fourier Transform Infrared (FTIR) spectra differentiating control and treatment soils. **(B)** The first three PCA-LDA loadings of the complete data set in the lower spectral region (≤1200 cm^-1^) showing the regions that discriminated across the two axis given in **(C)**. **(C)** Infrared spectra depicting the control and treatment soils, the spectral features have been annotated to indicate the different soil compounds relevant to the current study. An extension of this figure with complete annotations is given in the Supplementary Figure S1.

Fourier Transform Infrared-ATR offered insight into the properties of C compounds differentiating the control soils from those undergoing drought. We noted a clear separation of the control soils from those undergoing drought across the primary PCA-LDA component accounting for the majority of the variance (46%; **Figure 3A**). Plotting the first three PCA-LDA components (accounting for 73% of the variance) showed that the spectroscopic features responsible for separating the control soils from treatment soils were concentrated within the biochemical fingerprint region (<1250 cm^-1^) from which an identification of the compounds may be inferred (**Figure 3B**). A number of characteristic absorbance peaks were identified within this region (Supplementary Figure S1A). Peaks at 980–1000 and 1050–1100 cm^-1^ are likely carbohydrate and polysaccharide signatures, respectively (Solomon et al., 2005; Artz et al., 2008), and were significantly enriched (*p* = 0.01) in the soils from the drought treatments (**Figure 3C**). Conversely, peaks at 835 cm^-1^ (**Figure 3C**) and 1650 cm^-1^ (Supplementary Figure S1B) are lignin-like compounds (Artz et al., 2008), and were higher in the control soils compared with the soils undergoing drought (**Figure 3C**). Large peaks at 410 and at 500 cm^-1^ are signatures of metal-oxide bond stretches characteristic of clay and quartz minerals (Haberhauer et al., 1998) and were significantly higher in the control soils (*p* < 0.001).

### Excitation-Emission Matrix (EEM) Fluorescence Spectroscopy

**FIGURE 4:**
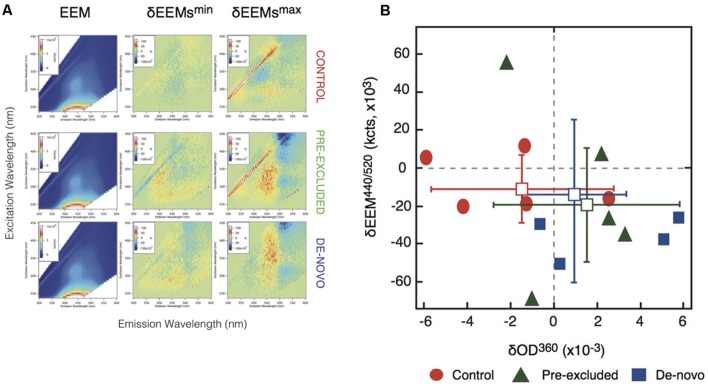
**Changes to carbon composition measured using Excitation-Emission matrix (EEM) spectroscopy. (A)** Scatter plots representing the correlations between changes in the optical absorption and emission data. Vertical axis is the difference in the EEM region centered at λ_ex_ = 440 nm and λ_em_ = 520 nm (i.e., the blue regions in **Figure 3A**). Horizontal axis is the difference in UV-vis absorption at 360 nm. **(B)** Excitation-emission matrix (EEM) analysis of control and excluded soils. Column 1 summarizes averaged EEM data for one replicate series of five extractions from each soil. Columns 2 and 3 report difference EEM (δEEM) data obtained by subtracting the averaged EEM from the control site from a selected individual EEM. Shown are the individual δEEM data that exhibited the smallest (column 2) and largest (column 3) differences from the averaged control. In-figure inserts show pixel intensity values (Column 1 = ×10^6^; Column 2 = ×10^3^).

To determine the properties of mineral-associated organic matter we performed EEM analysis of pyrophosphate extracts from control and drought soils. The spectra for the control and drought soils were similar (**Figure 4A**). However, a scatter plot showing EEM absorption/emission data of the δEEM^440/520^ region shows the soils undergoing drought were more similar to each other than either treatment was to the control samples within this region (**Figure 4B**). The separation between treatment and control soil plots was attributable, in part, to the putative loss of a long-wavelength emission associated with a spectral range representative of aromatic soil compounds within the treatment plots (Aiken, 2014; Supplementary Table S5). We interpret this as the further loss of chemically complex carbon compounds, in this case the aromatic moieties associated with fulvic acids, in the soils undergoing drought (**Figure 4A**).

### Soil Wet-up Experiments

**FIGURE 5:**
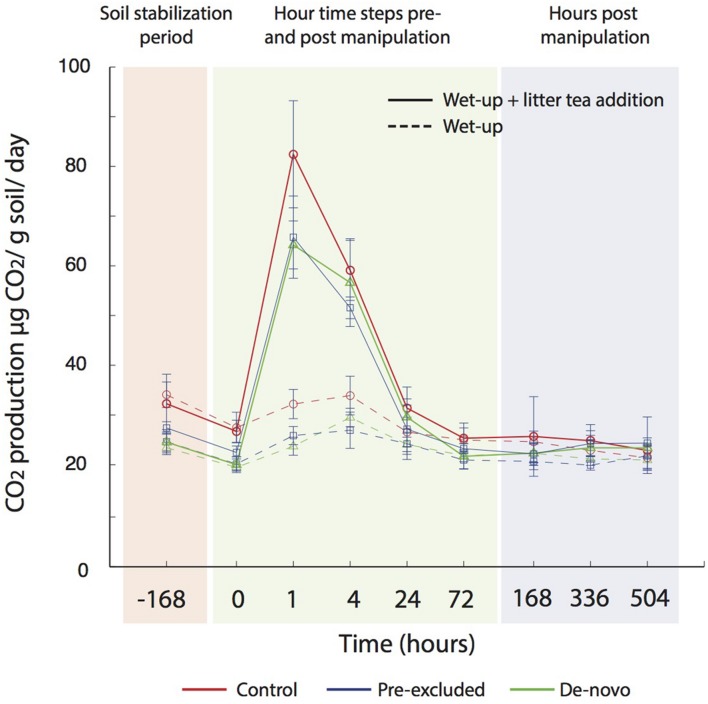
**Measured CO_2_ flux following wet up with and without additional substrate (leachate) addition.** A period of stabilization followed transfer of soils to mason jars (-168 h), prior to the addition water (± leachate), after which measurements were made in relatively high frequency over the first 3 days, and then measured once a day for 3 weeks.

Soils undergoing a year of drought were wet-up with (i) water and (ii) water ± leaf litter leachate (termed ‘litter tea’). Under both wet-up scenarios, CO_2_ production was higher in the control soils compared with soils undergoing drought (**Figure 5**). The magnitude of CO_2_ production was significantly higher under the litter tea addition, and maximal CO_2_ production was recorded earlier in the experiment (after 1 vs. 4 h for water addition only). CO_2_ production rates returned to pre-addition concentrations after approximately 24 h (**Figure 5**).

## Discussion

### Changing Composition and Structure of Dissolved and Particulate Organic Matter

In these tropical forest soils, prolonged drought fundamentally altered the composition and structure of dissolved and mineral-associated organic compounds. This was most prominent in the *de novo* soils, where we have previously documented broad changes in bacterial community composition (Bouskill et al., 2013), functional potential, osmolyte production, and enzyme activity following a modest decline in Ψ (Bouskill et al., submitted). Tandem mass spectrometry (metabolomics) found a significant increase in compounds related to metabolic biosynthesis and carbohydrate biosynthesis in treatment plots relative to control (**Figure 1**). Taken together, our data indicate that drought triggers a substantial metabolic response in the microbial community.

We sought to understand what impact this metabolic response had on the composition and stability of soil carbon. We had previously hypothesized (Bouskill et al., submitted) that the increased abundance of amino acids and carbohydrates was partly the result of organisms synthesizing and accumulating osmolytes in response to a decline in soil moisture. However, the increased abundance of compounds such as siderophores and antibiotics provided further cues to the environment experienced by soil microorganisms. Microbes in Fe-limited ecosystems commonly secrete siderophores to scavenge poorly soluble ferric-iron (Miethke and Marahiel, 2007). The overrepresentation of these compounds in the *de novo* soils supports our previous supposition that soluble Fe availability decreases under drought (Bouskill et al., 2013). This is also of significance for phosphorus availability in these soils (Chacon et al., 2006). Under drought conditions, as soils dry, oxygen permeates into the soil which can result in oxidation and precipitation of ferrous iron [Fe(II)] and co-precipitation of phosphorus and carbon (Kleber et al., 2015). Furthermore, redox oscillations, which would intensify under more frequent wet-dry cycles predicted for tropical regions (Dai, 2012), can alter the speciation and crystallinity of iron oxyhydroxides [Fe(OH)_3_; Hansel et al., 2005; Thompson et al., 2006; Yang et al., 2010], altering the retention of co-precipitated elements during reductive dissolution (Hansel et al., 2004). Strong correlations between Fe and P may partly explain a significant decline in P concentrations in extracts from soils undergoing drought, potentially exacerbating P limitation of tropical soils (Vitousek and Sanford, 1986). Given that P availability is an important driver of plant and microbe community activity (Jones and Lennon, 2010; Townsend et al., 2011), prolonged drought in tropical forests could further constrain a physiological response.

Of additional interest is a significant accumulation of fungal and Actinobacterial derived antibiotic compounds within the *de novo* soils. Our previous work reported an increased abundance of Actinobacteria under drought (Bouskill et al., 2013), which are known to induce secondary metabolism and antibiotic production under osmotic stress (Wong and Griffin, 1974; Bishop et al., 2004). Aside from the overall stimulation of the secondary metabolism, the elevated abundance of antibiotics under drought could originate from rapid changes in environmental conditions (e.g., water potential) that result in the establishment of organisms with drought tolerance traits that were previously uncompetitive or in low abundance in these typically moist soils. These rapid changes may select for antagonistic traits, including antibiotic production, that facilitate an organism’s competitiveness (Hibbing et al., 2010). Alternatively, there is growing evidence that antibiotics at sub-inhibitory concentrations act as signaling molecules facilitating intra- and interspecies communication (Romero et al., 2011). Relevant processes associated with this signaling mechanism include enhanced production of extracellular polysaccharides and biofilm formation (Bleich et al., 2015). We discuss the further evidence for and ecological significance of biofilm formation below.

Our data also support an often assumed but rarely observed (but see Cusack et al., 2011) coupling between microbial functional diversity and ecosystem processes, in this case enzyme potential activity. We have previously outlined two scenarios that might explain the observed increase in enzyme activity (Bouskill et al., submitted): (1) stabilization and/ or enhanced activity of enzymes sorbed onto mineral surfaces, and, (2) elevated enzyme production to support a physiological response to drought. While it is difficult to conclusively demonstrate that either is a dominant mechanism here, it is more likely that the latter scenario would lead to the changes in the composition of organic compounds noted with ATR-FTIR and EEMS. Specifically, the elevated activity of the cellulose degrading enzymes (Cellobiohydrolase and β-1,4-glucosidase) in soils undergoing drought could explain the lower abundance of *n*(C-O) bonds (∼1100 cm^-1^) indicative of cellulose (**Figure 3C**). Xylanase activity was also significantly higher in soils undergoing drought and there is some evidence that hemicellulose abundance was lower in these soils. Several peaks related to bond stretching or bending associated with hemicellulose compounds (e.g., 0-H stretch at 3403 cm^-1^, C-H bending at 1409 cm^-1^, and O-H bending at 1324 cm^-1^ (Peng et al., 2012), were lower within the soils undergoing drought relative to the controls (Supplementary Figure S1B). In light of this, we can conclude that hydrolytic enzyme activity, elevated in response to drought, was crucial in shaping observed changes in the carbon pool composition.

Experimental drought resulted in a significant decline in the relative intensity of bond stretches representing lignin (∼835 and 1650 cm^-1^; **Figure 3C**), again suggesting a higher rate of plant polymer decomposition. Further analysis using EEM also demonstrated the loss of a long wavelength emission associated with aromatic moieties of soil organic acids (**Figure 4B**). The loss of these compounds could result from elevated activity of peroxidase enzymes (Rezácová et al., 2007). We observed an increase in the abundance of genes related to the oxidative enzymes (Bouskill et al., submitted), which are widely distributed across bacterial taxa (Diaz et al., 2013) including the Actinobacteria (Le Roes-Hill et al., 2011), and taken together, our results are consistent with the notion that drought increases conditions of oxidative stress and the expression of oxidative enzymes targeting aromatic moieties (see Sinsabaugh, 2010 and references therein).

### Resistance of Pre-excluded Soils to Subsequent Drought

Tropical ecosystems are frequently assumed to possess a relatively low adaptive capacity to perturbation (Deutsch et al., 2008). This is due in many cases to lower seasonality, relative to temperate and high latitude environments, optimizing the performance of tropical organisms to a narrow climatic range and restricting the adaptive response to excursions from optimal conditions (Huey et al., 2009). However, a hysteretic response and selection for resistant genotypes can form the basis of an ecological response to future perturbation. The rapidity of this response depends on the turnover rate within a community, its genotypic diversity, and the rate of environmental change.

In the present study, soil physical and chemical measurements imply that the response of the pre-excluded soil plots to prolonged drought was moderate relative to the *de novo* soils, suggesting a physical conditioning of soils following the previous perturbation (1 year prior to the present study). We propose that the stress response of organisms to drought plays a role in this conditioning through physical re-structuring of soils (Crawford et al., 2012). In this case, polysaccharide production observed under drought is an important response of the pre-excluded communities, as reflected by the metabolomic (**Figure 2**) and ATR-FTIR data (**Figure 3**). Polysaccharide production was not as substantial in the *de novo* soils that were more sensitive to perturbation.

Biofilms, formed from the production of polysaccharides, are exuded by bacteria to provide protection from nutrient stress, redox changes and desiccation (Roberson and Firestone, 1992; Davey and O’toole, 2000; O’Toole and Stewart, 2005). In this case we propose that extracellular polysaccharides modify soil structure (Crawford et al., 2012), increase water holding capacity (Chenu, 1993; Rosenzweig et al., 2012), mitigating declines in Ψ, and reducing osmotic stress to communities undergoing subsequent periods of drought.

Despite an appreciation for the importance of biofilms in structuring microbial communities within the environment (Battin et al., 2003) and in aggregate formation important to the soil carbon cycle (Six et al., 2006; Tang et al., 2011), there has been little work in tropical forest soils that might support or refute our hypothesis. We contend that EPS production and biofilm formation is a response trait selected for as the community composition and abundance changes under declining water potential. However, a question remains as to whether this response trait can act as an effect trait and modify microbial activity at the ecosystem level, as has previously been reported for aquatic systems (Battin et al., 2003).

### Carbon Flux under Resumption of Throughfall

A central question in environmental microbiology asks whether perturbation restructures the functional traits and physiologies of microbial communities to an extent that impacts ecosystem greenhouse gas emissions (Schimel and Gulledge, 1998). The difficulty in addressing this question with respect to broad processes (e.g., CO_2_ production from respiration) arises because a significant fraction of the community can be considered functionally redundant, negating the ecological impacts of changes in community composition (particularly if changes are measured phylogenetically; Wertz et al., 2007), although there are studies that question this concept (Bell et al., 2005; Strickland et al., 2009; Cusack et al., 2011).

In this current study we sought to understand whether a cascade of drought-induced changes in community composition and activity that feed back on soil carbon composition also manifest at the ecosystem level through altered CO_2_ fluxes following wet-up. We previously hypothesized that the metabolic response to drought creates an intracellular C demand leading to increased extracellular enzyme production and mineralization of more complex organic compounds (Bouskill et al., submitted). This, combined with a release of intracellular solutes following cellular lysis (Halverson et al., 2000) could provide a larger pool of soluble C accessible for mineralization under wet-up leading to a larger CO_2_ pulse. This would be consistent with previous findings from lowland tropical forest soils (Cleveland et al., 2010), and further a field, for example within semi-arid grasslands (Placella et al., 2012) that show elevated CO_2_ production following the alleviation of drought, brought about through changes in substrate concentration (Cleveland et al., 2010), or O_2_ infusion stimulating microbial activity (Silver et al., 1999).

However, in the present study we observed a lower CO_2_ eﬄux from the soils previously exposed to drought after rewetting compared with control soils (**Figure 5**). A lower eﬄux of CO_2_ from some tropical under drought has been observed previously (Sotta et al., 2007; Wood and Silver, 2012), and seasonal data show strong positive correlations between precipitation and CO_2_ fluxes (Metcalfe et al., 2007). Wood and Silver (2012) recorded a 30% decrease in CO_2_ fluxes during a short-term (3-month) experimental drought, correlated with a decline in exchangeable P concentrations. They concluded that under drought microbial decomposition was P-limited, as noted for other tropical forests (Cleveland and Townsend, 2006). We also observed a significant decline in P concentrations under prolonged drought that would plausibly limit microbial activity. The functional gene data further demonstrates increased relative abundance of genes related to P limitation pathways within the soils undergoing drought (Bouskill et al., submitted).

Substrate availability could have led to reduced eﬄux of CO_2_ following rewetting. However, we believe this is unlikely as the qualitative trend in the data was consistent with or without the addition of substrate (in the form of a leaf litter leachate collected from the same forest), although substrate addition led to a higher CO_2_ eﬄux. In addition, communities in treatment plots mounted a metabolic response to drought that included carbon allocation to osmolyte and EPS production. Such a response would require significant carbon reserves inconsistent with the idea of substrate limitation in these soils (Manzoni et al., 2014). Substrate limitation of drought soils cannot be ruled out entirely, however. Communities under drought also invested carbon into the production of extracellular enzymes (Bouskill et al., submitted) that might indicate an increased demand for carbon that local substrate availability cannot fulfill.

Finally, changes in the biophysical and chemical properties of the soil matrix through EPS production could enhance the stability of soil communities (Chenu and Cosentino, 2011) and reduce susceptibility to osmotic shock. Indeed, the formation of biofilms in soils and sediments has previously been shown to alter ecosystem properties (Battin et al., 2003; Or et al., 2007; Holden, 2011). In the present study, cellular lysis due to alternating wet-dry cycles (Schimel et al., 2011) could be curtailed through population encasement in biofilms that could be responsible for the reduction in CO_2_ release observed. The impact of biofilm formation on the stability of these tropical soils following re-wetting, and the impact on availability of previously occluded soil carbon warrant further study.

## Conclusion

Despite only small changes in water potential, tropical soil microorganisms experienced osmotic stress and responded accordingly by producing compatible solutes, polysaccharides and secondary metabolites. They also produce more complex C degrading enzymes to fulfill an intracellular C demand (Supplementary Figure S2). The overall response to small fluctuations in water potential is a complex, hysteretic, biological, physical, and chemical response that ultimately results in a lower C eﬄux despite increased hydrolysis and oxidation of complex carbon compounds.

This hysteretic feed back is relevant for attempting to predict the response of belowground ecosystems in tropical forests to increasingly frequent periods of drought predicted to occur over the next century (Dai, 2012; Feng et al., 2013). However, it should be noted that tropical regions are characteristically heterogeneous in climate, soil moisture and belowground ecology. Further work is required to define a more pan-tropical response to drought that considers not only direct belowground responses but also the interactions and feed backs between above and belowground vegetation and the soil-microbial system to a changing climate.

## Author Contributions

NB performed the research, analyzed the results, ZY, HL, RB, BB, and ZH contributed to data collection and analysis. TN and H-YH contributed novel techniques for data collection. TW and WS, established the throughfall treatments in Puerto Rico. PN provided consultation for the work. EB, TW, and WS designed the research. NB and EB wrote the manuscript, with contribution from all co-authors.

## Conflict of Interest Statement

The authors declare that the research was conducted in the absence of any commercial or financial relationships that could be construed as a potential conflict of interest.

## Footnotes

1 http://www.massbank.jp/index.html?lang=en

2 http://www.genome.jp/kegg/
