# Supplementary material for: Belowground Response to Drought in a Tropical Forest Soil. II. Change in Microbial Function Impacts Carbon Composition
Source: Front Microbiol. 2016 Mar 15;7:323. doi: 10.3389/fmicb.2016.00323 (PMC4791749; doi:10.3389/fmicb.2016.00323)
Supplement: Supplementary file 1 [file Data_Sheet_1.DOCX]

**Supplemental Material**

**Belowground response to drought in a weathered tropical forest soil. II. Functional changes in microbial community impact the composition of soil carbon compounds**

Nicholas J Bouskill^a*^, Tana E Wood^b,c^, Richard Baran^d^, Zhao Hao^a^, Zaw Ye^a^, Ben P Bowen^d^, HsiaoChien Lim^a^, Peter S Nico^a^, Hoi-Ying Holman^a^, Benjamin Gilbert^a^, Whendee L Silver^g^, Trent R Northen^d^, & Eoin L Brodie^a,g*^

^a^Earth Sciences Division, Lawrence Berkeley National Laboratory, Berkeley, CA, 94720

^b^International Institute of Tropical Forestry, USDA Forest Service, Rio Piedras, PR, 00926.

^c^Fundación Puertorriqueña de Conservación, San Juan, PR, 00936

^c^Life Sciences Division, Lawrence Berkeley National Laboratory, Berkeley, CA, 94720.

^g^Department of Environmental Science, Policy and Management. University of California-Berkeley, Berkeley, CA, 94209.

*Corresponding authors:

Nicholas Bouskill, Ecology Department, Earth Sciences Division, Lawrence Berkeley National Laboratory, Berkeley, CA, 94702.

E-mail: [njbouskill@lbl.gov](mailto:njbouskill@lbl.gov)

Tel: (+1) 510-486-7490

Fax: (+1) 510-486-7152

Eoin Brodie, Ecology Department, Earth Sciences Division, Lawrence Berkeley National Laboratory, Berkeley, CA, 94720.

E-mail: elbrodie@lbl.gov

Tel: (+1) 510-486-6584

Fax: (+1) 510-486-7152

**Supplemental Materials and Methods**

*Throughfall exclusion:* This study was conducted in a humid tropical forest in the Bisley Research Watershed of the Luquillo Experimental Forest (LEF) in Puerto Rico (~ 350 m a.s.l; 18° 18N, -65° 50W). A detailed classification of these soils has been published previously (Silver et al., 1994). Briefly, these soils are classified as ultisols in the Humatus-Cristal-Zarzal series, and are derived from volcanic sediments with Tertiary-age quartz-diorite intrusions of the Rio Blanco stock. The soils are deep, clay rich and acidic, with high aluminum and iron content (Scatena, 1989). The throughfall exclusion experiment was established in a Tabonucco forest stand located on an upper ridge in June 2008 with an initial ten soil plots (Wood & Silver, 2012). A thorough description of the experiment has also been published previously (Bouskill et al., 2013). Throughfall was excluded from five of the soils with clear, corrugated plastic panels (1.54 m^2^) mounted 1 m above the forest floor at a 17° angle, for a period of 3 months before the shelters were removed and ambient throughfall resumed (these are the ‘pre-excluded’ soils in the present study). The five remaining soils were not sheltered and served as controls. The soils were not trenched to minimize soil disturbance. The following year (June, 2009), the shelters were replaced over these original five throughfall-excluded soils and five new (‘*de-novo’*) exclusion experiments were established (15 soil plots total). Soil samples (5–10g) were taken in triplicate from each soil plot at the outset of the study and again at 3 and 10 months following the placement of the throughfall shelters. Soil samples were shipped overnight to Berkeley at 4 °C, and the triplicate samples were composited in the laboratory.

The high soil clay content did not allow for direct centrifugation or filtration-based extraction of porewater. Therefore, soil water was extracted from 15 g of fresh soil by adding 15 ml ultra-pure water and vortexing for 10 minutes. Samples were filtered through a 0.2 μm nylon filter by centrifugation for 30 minutes at 2,500 rpm at 4 °C. The extracted soil water was used to measure pH, conductivity, cation and anion concentrations, water potential, and total organic carbon (TOC) as described previously (Bouskill et al., 2013), and also for osmolyte extraction (see below). The final volume of extracted soil water, minus the additional 15 mls, was taken as the soil water and measured cations, anions, and carbon concentrations were normalized to this volume and expressed as mM ml^-1^ of soil water (cations and anions) or mg L^-1^ (TOC). Fresh samples were also used for enzyme assays. Soil moisture was measured by drying 5 g of soil at 105 °C over 24 hours, the difference between the initial and final weights was taken as the soils water content. The remaining untouched soil was immediately frozen at -80 °C until nucleic acid extraction.

*Metabolite extraction:* To examine potential biological responses to changing water potential we reconstructed the metabolite profile in the dissolved organic fraction of the soil water after 10 months of throughfall exclusion. The vigorous nature of the extraction method likely disrupts and lyses cell walls effectively extracting both intracellular and extruded metabolites. Polar compounds were extracted by adding 1 ml methanol (-20 ^°^C) to the solution, vortexing for 30 s and incubating at -20 ^°^C for 3 min. Following incubation the solution was centrifuged for 1 minute at 2,350 g and 750 μl removed to a glass vial. Non-polar metabolites were extracted by adding 500 μl hot isopropanol (65 ^°^C) to the solution and incubating at 65 ^°^C for 3 minutes. The solution was centrifuged for 14,000 g and 750 μl removed to a glass vial. Both polar and non-polar supernatants were concentrated by spinvac and redissolved in 100 μl of methanol containing 1 μg ml^-1^ of 2-amino-3-bromo-5-methylbenzoic acid (ABMBA) as an internal standard. The samples were stored at 4 ^°^C, filtered through a 0.2 μm PVDF membrane microcentrifugal filter (National Scientific) and analyzed via LC-MS performed using normal phase liquid chromatography coupled to a quadrapole time-of-flight mass spectrometer. This method gave signal intensity and spectra data for a wide range of molecules (m/z range 52.08 - 1663.03) from each of the 15 samples. Spectra identification is described in the main text.

*Additional EEMs standardization methods*

Prior to analysis, the calibration of the excitation and emission monochromators were verified by scanning both the lamp output and the water Raman spectrum. The quantity of the sample added to the buffer was individually determined based on the UV-Vis data so that A^254^ = 0.2. EEMs were acquired using the photodiode detector with dark counts subtracted. The response of the detector was measured by acquiring a series of fluorescence emission intensities from quinine sulfate (QS) from 0 – 100 ppb using λ_ex_ = 310 nm and λ_em_ = 450 nm. These data were used to confirm the linearity of the detector response in the range relevant to the EEM spectra.

**Supplemental Results**

*Soil physicochemical response*: A detailed summary of the effect of experimental throughfall exclusion on the chemistry and bacterial diversity of these soils has been published previously (Bouskill et al., 2013). Throughfall exclusion reduced soil moisture and altered soil water chemistry (Table S1). Data from prolonged (10-month) throughfall exclusion resulted in lower soil moisture values for the pre-excluded (~65 % soil moisture) and *de-novo*soils (~60 %) compared to the control (~76 %) (Table S1). As previously reported (Bouskill et al., 2013), after the 10-month exclusion period aluminum (Al) and several redox sensitive compounds, e.g., iron (Fe) and molybdenum (Mo), in addition to phosphorus (P), were of significantly lower concentration in soil water extracts (Table S1). Soil Ψ was also significantly lower in soils undergoing throughfall exclusion (pre-excluded = -0.27 ± 0.06; *de-novo* = -0.34 ± 0.1 MPa) than the control soils (-0.19 ± 0.03 MPa), while sodium (Na) and potassium (K) concentrations in soil water were significantly higher (p = 0.01).

**Supplemental Tables**

Table S1: Table of physicochemical factors measured in April, 2010, 10 months following the placement of rainfall shelters.

.

Table S2: Permutational multivariate comparisons between biological data sets and physicochemical data. (a) Metabolite data vs Enzyme activities, and (b) Metabolite data Vs, select environmental data sets.. Abbreviations; Ψ = Water potential, Na = Sodium (a proxy for solute concentration. Significant relationships (*p* < 0.05) are highlighted by bold italics.

(a)

| **Sources of variance** | **D.f** | **S.S** | **F** | **R^2^** | ***p*** |
| --- | --- | --- | --- | --- | --- |
| **Beta-galactosidase** | **1** | **0.06** | **4.2** | **0.25** | **0.002** |
| ***Cellobiohydrolase*** | ***1*** | ***0.06*** | ***2.9*** | ***0.18*** | ***0.01*** |
| ***N-acetyl-D-glucosaminidase*** | ***1*** | ***0.05*** | ***2.9*** | ***0.23*** | ***0.004*** |
| ***Xylanase*** | ***1*** | ***0.06*** | ***3.8*** | ***0.23*** | ***0.005*** |

(b)

| **Sources of variance** | **D.f** | **S.S** | **F** | **R^2^** | ***p*** |
| --- | --- | --- | --- | --- | --- |
| ***Treatment*** | ***2*** | ***0.17*** | ***2.13*** | ***0.27*** | ***0.003*** |
| Ψ | 1 | 0.04 | 0.9 | 0.07 | 0.45 |
| ***Na*** | ***1*** | ***0.11*** | ***2.8*** | ***0.18*** | ***0.007*** |
| P | 1 | 0.06 | 1.3 | 0.09 | 0.2 |
| Fe | 1 | 0.05 | 1.16 | 0.08 | 0.28 |
| Treatment x Na x P | 2 | 0.08 | 1.4 | 0.12 | 0.27 |
| ***Na x P*** | ***1*** | ***0.08*** | ***2.5*** | ***0.14*** | ***0.03*** |
| Na x Fe | 1 | 0.04 | 1 | 0.06 | 0.42 |

Table S3: Mantel tests between different data sets. The phylogenetic data set was presented elsewhere (Bouskill et al., 2012). Significant relationships (*p* < 0.05) are highlighted by bold italics.

| **Matrix for comparison** | **Significance** | **Mantel r Statistic** |
| --- | --- | --- |
| ***Functional gene x Metabolite*** | ***0.02*** | ***0.5*** |
| ***Phylogenetic x Metabolite*** | ***0.04*** | ***0.61*** |
| Phylogenetic x Functional gene | 0.7 | -0.16 |

Table S4: Metabolites unambiguously identified through the KEGG database arranged by category. Values denote the average spectra peak height (± standard deviation).

Table S4 cont.

Table S5: Summary of literature attributions of EEM peaks (Aiken, unpublished data).

| **Peak Label** | **Excitation maximum (nm)** | **Emission maximum (nm)** | **Description of fluoropores** |
| --- | --- | --- | --- |
| B | 275 | 305 | Tyrosine-like |
| T | 275 | 340 | Tryptophan-like, |
| A | 260 | 400 – 460 | Humic-like |
| M | 290 – 310 | 370 – 410 | Marine humic-like |
| C | 320 – 360 | 420 – 460 | Humic-like |
| D | 390 | 509 | Soil fulvic acid |
| E | 455 | 521 | Soil fulvic acid |
| N | 280 | 370 | Plankton derived |

Figure S1: (a) FTIR spectra from the control sample overlaid with the principal descriptive IR adsorption bands. Superscript numbers denote references used to identify bands. Reference numbers: 12: Solomon et al., 2005; 13: Kačuráková and Mathlouthi, 1996; 14: Artz et al., 2008. 15: Haberhauer et al., 1998. (b) Comparative spectra for control and treatment soils. Data shown is the difference between treatment and control soils that highlights some of the conclusions from the text.

(a)

(b)

Figure S2: Conceptual model highlighting the differences in soil physical and biological properties at the pore scale between soils experiencing throughfall-exclusion (b) relative to control conditions (a). The soil pore is surrounded by different minerals, including goethite, and under saturated conditions (a) solutes are diffuse, P and Fe(II) available and microbial communities use aerobic or dissimilatory anaerobic pathways in respiration of both LMW and complex C-sources. (b) Upon throughfall-exclusion, a reduction in porewater volume occurs, constraining substrate diffusion, concentrating solutes. Oxygen incursion increases and reduces the availability of both P and Fe(II), through Fe(II) reoxidation and inhibition of Fe(III) reduction. Fe(III) can complex P (and LMWC) on mineral surfaces. Microbial communities change in response and microorganisms themselves are osmotically and oxidatively stressed, and limited by nutrients (mainly P in this case). In response, resource allocation directs internal C stores and easily acquirable LMWC from DOC to maintain cellular integrity and preserve cellular metabolism. However, this process imposes a higher C-demand on the community and, upon limitation by LMWC, microbes increase production of extracellular enzymes to hydrolyze organic matter and access additional C to satisfy the increased demand.

(a)

(b)
